# Label-Free Fluorescence Assay of S1 Nuclease and Hydroxyl Radicals Based on Water-Soluble Conjugated Polymers and WS_2_ Nanosheets

**DOI:** 10.3390/s16060865

**Published:** 2016-06-13

**Authors:** Junting Li, Qi Zhao, Yanli Tang

**Affiliations:** Key Laboratory of Analytical Chemistry for Life Science of Shaanxi Province, Key Laboratory of Applied Surface and Colloid Chemistry, Ministry of Education, School of Chemistry and Chemical Engineering, Shaanxi Normal University, Xi’an 710062, China; lijunting@snnu.edu.cn (J.L.); lunar0315@163.com (Q.Z.)

**Keywords:** S1 nuclease, hydroxyl radicals, fluorescent sensing, label-free, conjugated polymers, WS_2_ nanosheet

## Abstract

We developed a new method for detecting S1 nuclease and hydroxyl radicals based on the use of water-soluble conjugated poly[9,9-bis(6,6-(*N,N,N*-trimethylammonium)-fluorene)-2,7-ylenevinylene-co-alt-2,5-dicyano-1,4-phenylene)] (PFVCN) and tungsten disulfide (WS_2_) nanosheets_._ Cationic PFVCN is used as a signal reporter, and single-layer WS_2_ is used as a quencher with a negatively charged surface. The ssDNA forms complexes with PFVCN due to much stronger electrostatic interactions between cationic PFVCN and anionic ssDNA, whereas PFVCN emits yellow fluorescence. When ssDNA is hydrolyzed by S1 nuclease or hydroxyl radicals into small fragments, the interactions between the fragmented DNA and PFVCN become weaker, resulting in PFVCN being adsorbed on the surface of WS_2_ and the fluorescence being quenched through fluorescence resonance energy transfer. The new method based on PFVCN and WS_2_ can sense S1 nuclease with a low detection limit of 5 × 10^−6^ U/mL. Additionally, this method is cost-effective by using affordable WS_2_ as an energy acceptor without the need for dye-labeled ssDNA. Furthermore, the method provides a new platform for the nuclease assay and reactive oxygen species, and provides promising applications for drug screening.

## 1. Introduction

The detection of endonuclease activity is essential because endonuclease plays an important role in many biological processes. Endonuclease has been widely used as a tool to remove non-annealed polynucleotide tails and hairpin loops in RNA and DNA duplexes, molecular cloning, and gene analysis [1,2,3]. S1 nuclease, a well-known multifunctional endonuclease, can hydrolyze ssDNA or RNA into 5’-phosphomononucleotide and 5’-phosphooligonucleotide and is employed as a model system. S1 nuclease has been particularly used to probe the disruption of the DNA structure by numerous carcinogens and antimitotic drugs [4]. Numerous methods based on advanced materials have been developed to detect S1 nuclease, such as colorimetric, electrochemical, and fluorescent assays [5,6,7,8,9,10]. However, most of these methods have some drawbacks, such as low detection limits, high cost and/or time-consuming assay procedures.

Hydroxyl radicals are highly reactive in many biological processes and can damage virtually all types of macromolecules, including carbohydrates, nucleic acids, lipids, and amino acids [11,12,13,14]. Hydroxyl radicals have received increasing attention due to their function in mutagenesis, carcinogenesis, and aging [15,16,17,18]. The DNA damage caused by hydroxyl radicals generates characteristic mutagenic base lesions and strand fragments in cellular systems [15,19]. To monitor DNA damage caused by hydroxyl radicals, methods based on the FRET technique were established [7,9,20,21,22]. Nevertheless, considering the crucial roles of S1 nuclease and hydroxyl radicals in many biological events, it is important to develop simple and convenient strategies for their analysis. 

Recently, single- and few-layered two-dimensional (2D) transition metal dichalcogenides (TMDCs), as planar covalent-network solids, have attracted growing attention in the fields of electronics, sensors, optics, catalysis, and energy harvesting, due to their special structures with high specific surface area and remarkable electronic properties [23,24,25]. Layered tungsten disulfide (WS_2_), one of the newly emerging 2D TMDCs, which consists of S−W−S sandwiches in a trigonal prismatic coordination, has been recognized as a novel nanomaterial in biomedical applications [26,27,28] because the large lateral dimensions and high surface areas of layered WS_2_ can effectively quench tagged fluorophores [29]. However, few bioassays based on WS_2_ nanosheets have been developed [29,30,31,32,33]. Thus, it is of interest to explore their new application as a biosensing platform.

Various conjugated polymer materials, especially water-soluble conjugated polymers (WSCPs), have been studied widely as useful platforms for sensitive biosensors and chemosensors [34,35,36,37]. WSCPs are composed of a large number of conjugated repeated units, which provides strong absorption and emission in the Ultraviolet−Visible light (UV–vis) range. When WSCPs are excited, the excitation energy along the main chain can rapidly transfer to an energy or electron acceptor, which accounts for the fluorescence signal amplification [35,36]. Thus, sensors based on WSCPs provide sensitive and specific platforms for various targets, such as oligonucleotides, enzymes, proteins, inhibitors, and toxic metal ions [7,37,38,39,40,41,42,43,44]. However, to the best of our knowledge, WSCPs and WS_2_ nanosheets used as a sensing platform have not been reported.

In this paper, we design a new approach to detect the activity of the S1 nuclease and hydroxyl radicals based on water-soluble PFVCN and WS_2_ nanosheets. In our strategy, PFVCN can be absorbed on the surface of single-layer WS_2_ nanosheets via electrostatic interactions, and WS_2_ nanosheets act as an efficient quencher for PFVCN [29]. When ssDNA is added, the fluorescence of PFCVN is recovered to some extent because of the much stronger electrostatic interaction between PFVCN and the ssDNA probe, leading to PFVCN leaving the surface of WS_2_. Upon addition of S1 nuclease or hydroxyl radicals, the ssDNA probe is hydrolyzed into small fragments, and PFVCN is adsorbed on the nanosheets, resulting in the fluorescence of PFVCN being quenched. Thus, S1 nuclease activity and hydroxyl radicals can be measured by monitoring the PFVCN fluorescence intensity, which is cost-effective, sensitive and label-free without the need for dye labeling of the ssDNA. This platform composed of WSCPs and WS_2_ also provides a new sensing system for biosensors.

## 2. Materials and Methods

### 2.1. Materials and Measurements

The oligonucleotides, ATP and BSA were purchased from Shanghai Sangon Biological Engineering Technology & Service Co. Ltd. (Shanghai, China). S1 nuclease and thiourea were obtained from Sigma (St. Louis, MO, USA). EcoRI, Exonuclease III and Klenow fragment polymerase (KF polymerase) were purchased from Dalian Takara Biotechnology Co. Ltd. (Dalian, China). The monolayer tungsten disulfide (WS_2_) nanosheet was purchased from Nanjing XFNano Material Tech. Co. Ltd. (Nanjing, China) Cationic poly[9,9-bis(6,6-(*N,N,N*-trimethylaminium)-fluorene)-2,7-ylenevinylene-co-alt-2,5-dicyano-1,4-phenylene] (PFVCN) was synthesized according to the literature [45]. The oligonucleotide sequence used in our experiments was as follows: 5′-CAA TGG AAC TAT TCG GCA TCA ATA CTC ATC-3′. The concentration of oligonucleotide was determined by measuring the absorbance at 260 nm in a 250 μL quartz cuvette. UV−vis absorption spectra were taken on a Lambda 35 spectrophotometer (Perkin Elmer, Waltham , MA, USA). The fluorescence spectra were recorded on a F-7000 spectrophotometer (Hitachi, Hitachi, Tokyo, Japan) equipped with a xenon lamp excitation source. The zeta potentials were measured on a Nano-ZS90 (Malvern Instruments, Malvern, UK). All solutions were prepared with ultrapure water from a Millipore filtration system.

### 2.2. Procedure

#### 2.2.1. Enzyme Assay

Forty microliters of the mixture containing 10 nM ssDNA probe and various concentrations of S1 nuclease was incubated in an enzyme reaction buffer (60 mM CH_3_COONa, 200 mM NaCl, and 2 mM Zn(SO_4_)_2_, pH 4.6) at 37 °C for 0.5 h. The mixture was heated at 95 °C for 10 min to terminate the cleavage reaction. Then, the reaction solution was diluted with Tris-HCl buffer (20 mM, pH 7.4) before the addition of WS_2_ nanosheets (1 μg/mL). After incubating the mixture for 10 min at 25 °C, PFVCN solution (10^−6^ M in repeat units) was added to the reaction with a final volume of 2 mL. The final concentration of S1 nuclease ranged from 5.0 × 10^−6^ U/mL to 0.7 U/mL. The emission spectra were measured at an excitation wavelength of 470 nm at 25 °C.

#### 2.2.2. Assay for S1 Nuclease as a Function of Incubating Time

A series of reaction mixtures containing a fixed concentration of ssDNA probe and S1 nuclease were incubated at 37 °C for 0, 5, 10, 15, 20, 25, 30, and 35 min, respectively. The subsequent experimental procedure was the same as the enzyme assay.

#### 2.2.3. Inhibition Assay by ATP

For enzyme inhibition experiments, a mixture containing 0.5 U/mL S1 nuclease and various concentrations of ATP ranging from 0 to 70 μM was incubated before the addition of the ssDNA probe. The subsequent procedure was the same as the enzyme assay. 

#### 2.2.4. Specificity Assay of S1 Nuclease

To verify the specificity of S1 nuclease, ExoIII, EcoRI, BSA and KF polymerase were used to replace S1 nuclease. According to the aforementioned experimental procedure, the fluorescence spectra were measured under the same conditions.

#### 2.2.5. ·OH assay and Inhibition Assay by Thiourea

In 2.0 mL 20 mM Tris-HCl buffer (pH 7.0), the solution of the ssDNA probe (10 nM) was treated with various concentrations of Fenton’s agent ([Fe^2+^]:[H_2_O_2_]:[DTT] = 1:10:10) for 5 min before the addition of WS_2_ nanosheets (1 μg/mL). After incubating the mixture for 10 min at 25 °C, the PFVCN solution (10^−6^ M) was added. The concentration of hydroxyl radicals was determined according to Fe^2+^, which ranged from 0.01 μM to 5 μM. The emission spectra were measured with the excitation wavelength of 470 nm at 25 °C. The inhibition experiments were the same as in the above procedure, except for the involvement of thiourea in the reaction solution before the addition of the ssDNA probe.

## 3. Results and Discussion

### 3.1. Array Mechanism

**Scheme 1:**
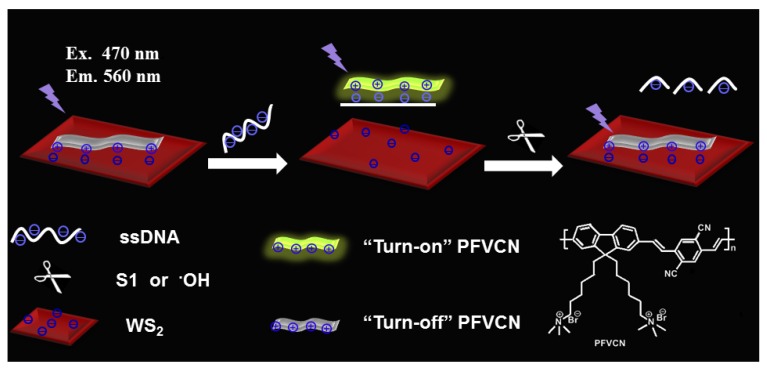
Proposed Strategy for the Hydrolysis of ssDNA by S1 Nuclease or Hydroxyl Radicals and the Chemical Structure of PFVCN

The proposed principle of the detection of S1 nuclease and hydroxyl radicals is illustrated in Scheme 1. Water-soluble cationic PFVCN is used as the optical transducer in the biosensor, and WS_2_ is used as a fluorescence quencher. WS_2_ nanosheets were purchased commercially with the thickness of ~1.0 nm and the size in the range of 20–500 nm, which was provided by the supplier. Also, we obtained the TEM image of WS_2_ that is coincident with data from the supplier (Figure S1). The PFVCN can be absorbed on the surface of single-layer WS_2_ nanosheets via electrostatic interactions. According to the information provided by the supplier, the WS_2_ was prepared through a controllable lithiation process leading to the WS_2_ having a negative charge [46] (the ζ potential of WS_2_ was measured as −26.0 ± 0.6 mV), resulting in substantial fluorescence quenching through fluorescence resonance energy transfer [32,47,48,49]. Upon the addition of ssDNA, PFVCN can form complexes with ssDNA due to the stronger electrostatic interaction between them. PFVCN then leaves the surface of the WS_2_ nanosheets and emits an enhanced fluorescence signal. When ssDNA is cleaved by S1 nuclease or ·OH into small fragments, the PFVCN/ssDNA complexes do not form because of their weaker affinity, and PFVCN is adsorbed on the nanosheets, causing the fluorescence of PFVCN to be quenched. Figure S2a shows that the PFVCN fluorescence change is coincident with the proposed principle. Control experiments show that S1 nuclease and hydroxyl radicals have no obvious effect on the PFVCN fluorescence intensity in the presence or absence of WS_2_ (Figure S2b,d). Also, the fluorescence intensity of PFVCN keeps stable in the presence of ssDNA without WS_2_ (Figure S2c). Consequently, the changes of the fluorescence intensity of PFVCN can be used to detect S1 nuclease and OH and to sense the hydrolysis of ssDNA without the need for a label on the ssDNA.

### 3.2. Optimization of the Experimental Conditions

**Figure 1:**
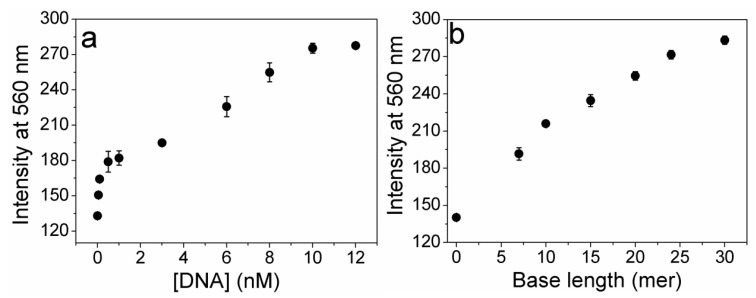
(**a**) Fluorescence intensity of PFVCN/ssDNA in Tris-HCl buffer solution (20 mM, pH 7.4) with different concentrations. [ssDNA] = 0−12 nM; (**b**) Fluorescence intensity of PFVCN/ssDNA in Tris-HCl buffer solution (20 mM, pH 7.4) with different base lengths. [PFVCN] = 1.0 × 10^−6^ M, [ssDNA] = 10 nM, [WS_2_] = 1 μg/mL. The error bars represent the standard deviations of three parallel measurements. The excitation wavelength is 470 nm.

To study the effect of the concentration and length of the nucleotide acids on the degree of fluorescence recovery, we investigated the fluorescence intensity of PFVCN (1.0 × 10^−6^ M in RUs) in the presence of the ssDNA probe in different concentrations. As shown in Figure S3a, WS_2_ nanosheets have a maximum absorption at 258 nm and emit very weak fluorescence with the excitation wavelength at 258 nm. The concentration of WS_2_ was optimized first in the presence of 1.0 × 10^−6^ M PFVCN. When the concentration of WS_2_ is 1 μg/mL, the fluorescence intensity of PFVCN is quenched greatly and reaches the plateau (Figure S3b). Thus, 1.0 × 10^−6^ M PFVCN and 1 μg/mL WS_2_ were used in following experiments. Furthermore, the control experiments also showed that the fluorescence of PFVCN/WS_2_ system was not interfered by S1 and hydroxyl radicals (Figure S2c). The effect of ssDNA concentration on PFVCN/WS_2_ system fluorescence was shown in Figure 1a. The fluorescence intensity gradually increased with the increase of the ssDNA probe concentration, resulting from the increased number of PFVCN complexes with ssDNA leaving the surface of WS_2_. When the concentration of ssDNA was increased to 10 nM, the fluorescence intensity of PFVCN reached a plateau. To investigate the effect of the nucleotide acid base length on the fluorescence enhancement, ssDNA (10 nM) with different base lengths, varying from 7- to 30-mer, were tested. As shown in Figure 1b, the fluorescence intensity of PFVCN gradually increased with the increase of the ssDNA length due to the stronger electrostatic interaction between PFVCN and the longer ssDNA. When the length of ssDNA was a 30-mer, the fluorescence of PFVCN reached a maximum; however, the rate of fluorescence increase became slower. These results indicate that the fluorescence enhancement of PFVCN is dependent on concentration and length of the ssDNA. Accordingly, 30-mer ssDNA at a concentration of 10 nM was chosen for the following analytical studies. This method is cost-effective due to use of cheap WS_2_ as an energy acceptor without the need for dye-labelling the ssDNA.

### 3.3. Sensing of S1 Nuclease

**Figure 2:**
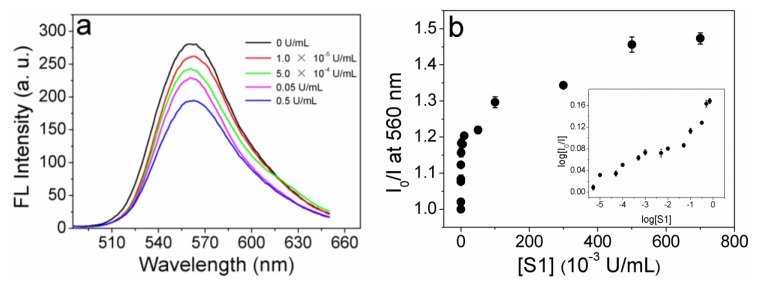
(**a**) Fluorescence emission spectra of PFVCN/ssDNA in Tris-HCl buffer solution (20 mM, pH 7.4) with the addition of S1; (**b**) Ratio of PFVCN fluorescence intensity with increasing amount of S1 (Insert: plots of log[I_0_/I] *vs.* log[S1]). [PFVCN] = 1.0 × 10^-6^ M, [ssDNA] = 10 nM, [WS_2_] = 1 μg/mL. The error bars represent the standard deviations of three parallel measurements. The excitation wavelength is 470 nm.

We then investigated the detection of S1 by measuring the fluorescence spectra under various concentrations of S1. The final concentration in the samples ranged from 0 to 0.7 U/mL. As shown in Figure 2a, the emission of PFVCN decreases with the increasing concentration of S1 from 0 to 0.5 U/mL. The ssDNA substrate was cleaved into small fragments by S1, which reduced the interactions between PFVCN and ssDNA and prompted the adsorption of PFVCN on the WS_2_ surface. These results are consistent with the results in Figure 1b. Figure 2b demonstrates the relationship of the fluorescence intensity ratio (I_0_/I) of PFVCN to the concentration of S1, where I_0_ and I are the fluorescence intensities of sensor solution in the absence and presence of S1 nuclease, respectively. The ratio increases with increasing of S1 concentration, which means the fluorescence of PFVCN decreases due to quenching by WS_2_ upon ssDNA analysis by S1. The detection limit was 5 × 10^−6^ U/mL (the signal at the detection limit (S_dl_) is given by: S_dl_ = S_bl_ − 3 * σ_bl_, where S_bl_ is the signal for a blank without S1, σ_bl_ is the known standard deviation for the blank’s signal from 11 experiments. In this case, S_dl_, S_bl_, and σ_bl_ are 275, 281, and 1.5, respectively.), which is lower than that reported previously by Zhang *et al.* ( 5 × 10^−5^ U/mL) [50], Yuan *et al.* (1.4 × 10^−6^ U/μL) [51], Zhou *et al.* (0.04 U/mL) [10] and Chu *et al.* (5 × 10^−7^ U/μL) [52]. He *et al.* studied the interaction between ssDNA and graphene oxide and developed a GO based biosensor for S1 nuclease with the detection limit of 5.8 × 10^−4^ U/mL [53], which is also higher than that obtained in this work. When the S1 concentration was 0.5 U/mL, the ratio reached a plateau and did not increase further, which means the ssDNA probe was cleaved completely by S1.

**Figure 3:**
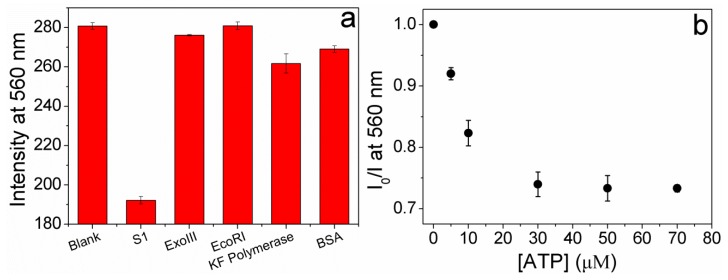
(**a**) Specificity of the assay toward other enzymes. [PFVCN] = 1.0 × 10^−6^ M, [WS_2_] = 1μg/mL, [ssDNA] = 10 nM, [S1] = [EcoRI] = 0.5 U/mL, [KF Polymerase] = [ExoIII] = 5 U/mL, [BSA] = 0.01 mg/mL; (**b**) Inhibition efficiency of S1 nuclease by ATP in Tris-HCl buffer solution (20 mM, pH 7.4). [PFVCN] = 1.0 × 10^−6^ M, [WS_2_] = 1 μg/mL, [S1] = 0.5 U/mL, [ssDNA] = 10 nM. The error bars represent the standard deviations of three parallel measurements. The excitation wavelength is 470 nm.

To study the selectivity of this method, we measured the fluorescence spectra of four enzymes, including ExoIII, EcoRI, BSA and KF polymerase, under the same experimental conditions as the S1 measurement. As shown in Figure 3a, nearly negligible fluorescence change was observed in the presence of ExoIII, EcoRI, BSA and KF polymerase relative to the control experiment (without any enzyme). The fluorescence intensity of PFVCN did not decrease dramatically after incubation with the other four enzymes, which indicates that ssDNA cannot be cleaved by these enzymes. These results indicate that the method is specific for S1.

Furthermore, S1 reaction time was optimized. A series of reaction mixtures containing a fixed concentration of 10 nM ssDNA probe and 0.5 U/mL S1 nuclease was incubated at 37 °C for 0, 5, 10, 15, 20, 25, 30, or 35 min. As shown in Figure S4, the fluorescence intensity of PFVCN gradually decreased as a function of time. The fluorescence intensity rapidly decreased during the first 5 min. The fluorescence intensity remained almost constant after 25 min, which demonstrates that the S1 nuclease detection can be completed in a short time.

In addition, we further studied the inhibition of the enzymatic activity of S1. ATP, a well-known S1 nuclease inhibitor, was used to inhibit the enzymatic activity of S1. The concentration of S1 was fixed at 0.5 U/mL. Figure 3b shows the inhibition efficiency of ATP varied in the concentration range from 0 to 70 μM (I_0_ and I are the fluorescence intensities of the sensor solution in the absence and presence of ATP). The activity of S1 was inhibited by the addition of 30 μM ATP. The results demonstrate that the developed method demonstrates strong performance not only in the assay of endonucleases activity but also in the screening of endonucleases inhibitors, which is of great importance in modern drug discovery. 

### 3.4. Sensing of Hydroxyl Radicals

**Figure 4:**
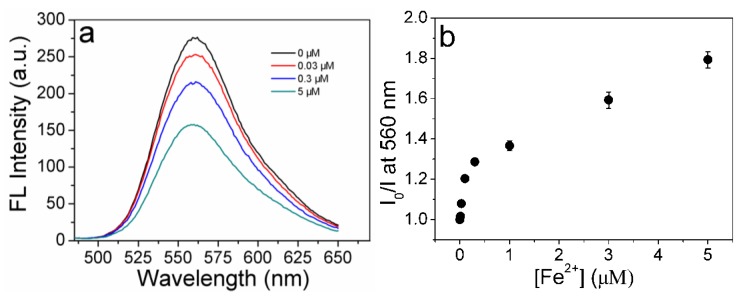
(**a**) Fluorescence emission spectra of PFVCN/ssDNA in Tris-HCl buffer solution (20 mM, pH 7.0) with the addition of Fenton reagent ([Fe^2+^]:[H_2_O_2_]:[DTT] = 1:10:10); (**b**) Ratio of PFVCN fluorescence intensity with increasing amount of Fe^2+^. [PFVCN] = 1.0 × 10^−6^ M, [ssDNA] = 10 nM, [WS_2_] = 1 μg/mL. The error bars represent the standard deviations of three parallel measurements. The excitation wavelength is 470 nm.

Hydroxyl radicals can damage virtually all types of macromolecules, such as carbohydrates, nucleic acids and lipids; this damage is highly dangerous and toxic to organisms. Assays for DNA cleavage by hydroxyl radicals can be performed by using a similar approach with the addition of Fenton’s reagent (Fe^2+^ + H_2_O_2_). Fenton’s reagent generates hydroxyl radicals that randomly cut DNA into different sequence fragments and even single bases. Fenton’s reagent ([Fe^2+^]:[H_2_O_2_]:[DTT] = 1:10:10) was chosen here to generate hydroxyl radicals. To study the activity of hydroxyl radicals, we measured the fluorescence spectra in 20 mM Tris-HCl buffer at pH 7.0 ([PFVCN] =1.0 × 10^−6^ mol/L in RUs, [ssDNA] = 1.0 × 10^−8^ mol/L, [WS_2_] = 1 μg/mL) at various concentrations of Fe^2+^ (from 0.01 μM to 5 μM). Figure 4a shows that the fluorescence of PFVCN gradually decreases with the increasing of Fe^2+^ concentration, which results from the nucleotide acids being cleaved by hydroxyl radicals and gradually being reduced by Fenton’s reagent. Figure 4b shows that the fluorescence intensity ratio (I_0_/I) of PFVCN increases with the increasing of Fe^2+^ concentration, where I_0_ and I are the fluorescence intensities of the sensor solution in the absence and presence of Fenton’s reagent, respectively. The concentration of hydroxyl radicals was related to Fe^2+^, and the detection limit was 0.01 μM. The result indicates that our new platform also can be used to detect hydroxyl radicals sensitively and simply.

Some antioxidants display effective capabilities to scavenge ·OH; therefore, they are able to inhibit the cleavage of DNA by OH. Thiourea was selected to clean up hydroxyl radicals in this case. As shown in Figure S5, the inhibition efficiency of thiourea varied in the concentration range from 0 to 0.7 mM, whereas the concentration of Fe^2+^ was fixed at 5 μM (I_0_ and I are the fluorescence intensities of the sensor solution in the absence and presence of thiourea). The activity of ·OH was effectively inhibited at 0.5 mM. Therefore, the developed platform provides a rapid and convenient method to screen anti-oxidant natural products or drugs.

## 4. Conclusions

In summary, we have designed a new method to detect S1 nuclease and hydroxyl radicals by taking advantage of the new platform based on PFVCN and WS_2_ nanosheets. This strategy has three significant characteristics. First, it is sensitive with a detection limit of S1 5 × 10^−6^ U/mL, which is far lower than reported in the literature. Second, the assay is cost-efficient without the need for dye-labelling of ssDNA due to the use of economic WS_2_ as the acceptor. Third, this method is simple and rapid, avoiding complicated washing and separation procedures. In virtue of these advantages, the proposed strategy combining water-soluble conjugated polymer and WS_2_ nanosheets provides new insight in the area of biosensors for endonuclease detection and the sensing of other reactive oxygen species.

## Supplementary Materials

The following are available online at http://www.mdpi.com/1424-8220/16/6/865/s1, Figure S1: TEM image of WS_2_, Figure S2: The fluorescence spectra of PFVCN/ssDNA/WS_2_, PFVCN/ssDNA/ S1/WS_2_, PFVCN/ssDNA , PFVCN/ssDNA/OH/WS_2_, Figure S3: Absorption and emission spectra of WS_2_, and PFVCN/WS_2_ in Tris-HCl buffer solution, Figure S4: Fluorescence intensity of PFVCN/ssDNA in the presence of S1 nuclease incubated for different periods in Tris-HCl buffer solution, Figure S5: Inhibition efficiency of hydroxyl radical by thiourea in Tris-HCl buffer solution.
