# Supplementary material for: Label-Free Fluorescence Assay of S1 Nuclease and Hydroxyl Radicals Based on Water-Soluble Conjugated Polymers and WS2 Nanosheets
Source: Sensors (Basel). 2016 Jun 13;16(6):865. doi: 10.3390/s16060865 (PMC4934291; doi:10.3390/s16060865)
Supplement: Supplementary file 1 [file sensors-16-00865-s001.pdf]

Sensors **2016**, *16*, x

Supplementary Materials: Label-Free Fluorescence Assay of S1 Nuclease and Hydroxyl Radicals Based on Water-Soluble Conjugated Polymers and WS_2 Nanosheets

Junting Li, Qi Zhao and Yanli Tang

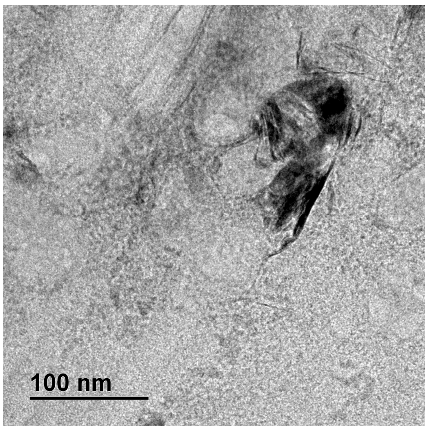

Figure S1. The TEM image of WS_2 nanosheets.

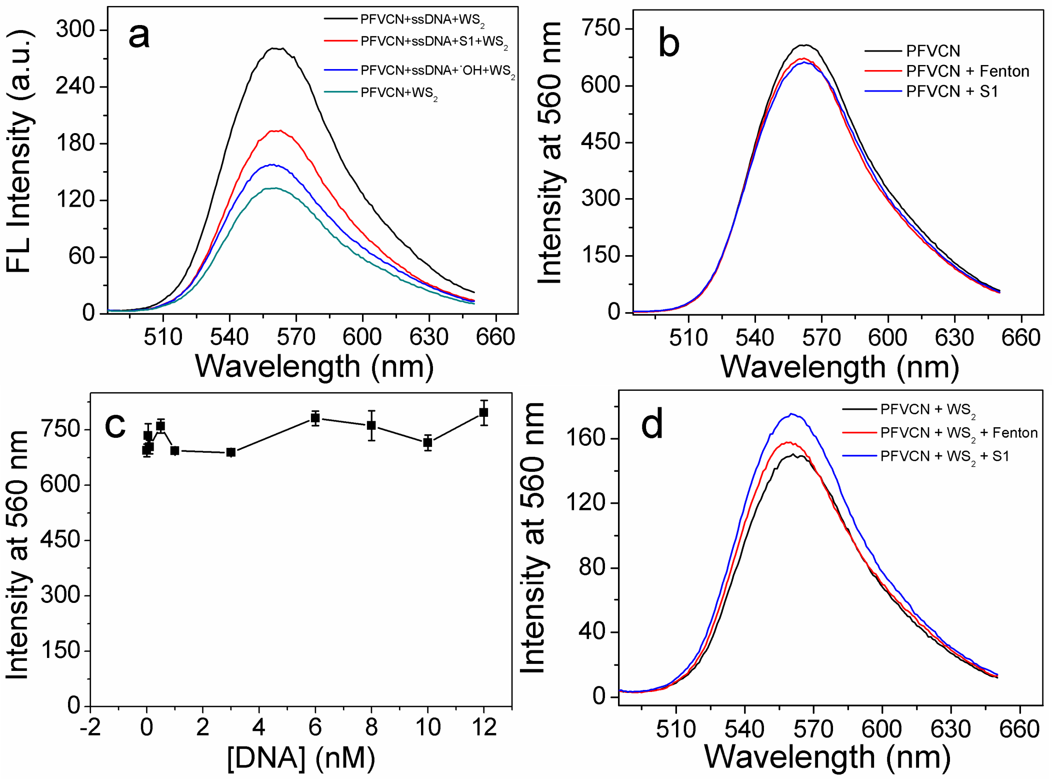

Figure S2. (**a**) The fluorescence spectra of PFVCN/ssDNA/WS_2, PFVCN/ssDNA/ S1/WS_2, PFVCN/ssDNA /·OH/WS_2 and PFVCN/WS_2 in Tris-HCl buffer solution (20 mM，pH 7.4); (**b**) The fluorescence intensity of PFVCN in the presence of S1 or Fenton reagent; (**c**) The fluorescence intensity of PFVCN in the presence of ssDNA; (**d**) The fluorescence intensity of PFVCN/WS_2 in the presence of S1 or Fenton reagent. [PFVCN] = 1.0×10^{-6} M, [WS_2] = 1 μg/mL, [S1] = 0.5 U/mL, [Fe^{2+}] = 5 μM. The excitation wavelength is 470 nm.

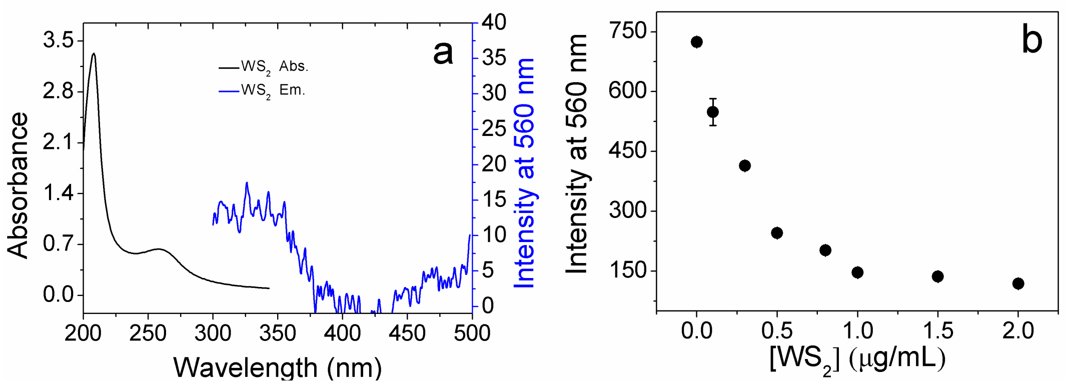

Figure S3. (**a**) The absorption and emission spectra of WS_2; (**b**) The fluorescence intensity of PFVCN in the presence of WS_2 in Tris-HCl buffer solution. [PFVCN] = 1.0 × 10^{-6} M, [S1] = 0.7 U/mL, [Fe^{2+}] = 5 μM. The error bars represent standard deviations three parallel measurements. The excitation wavelength is 470 nm.

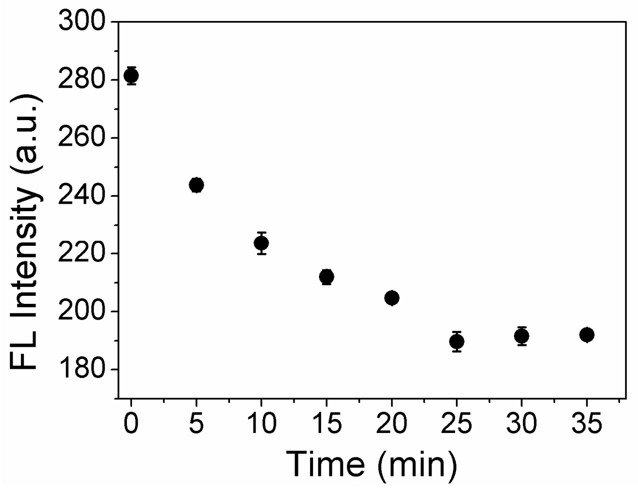

Figure S4. Fluorescence intensity of PFVCN/ssDNA in the presence of S1 nuclease incubated for different periods in Tris-HCl buffer solution (20 mM, pH 7.4). The error bars represent standard deviations three parallel measurements. [PFVCN] = 1.0 × 10^{-6} M, [WS_2] = 1 μg/mL, [S1] = 0.5 U/mL, [ssDNA] = 10 nM. The excitation wavelength is 470 nm.

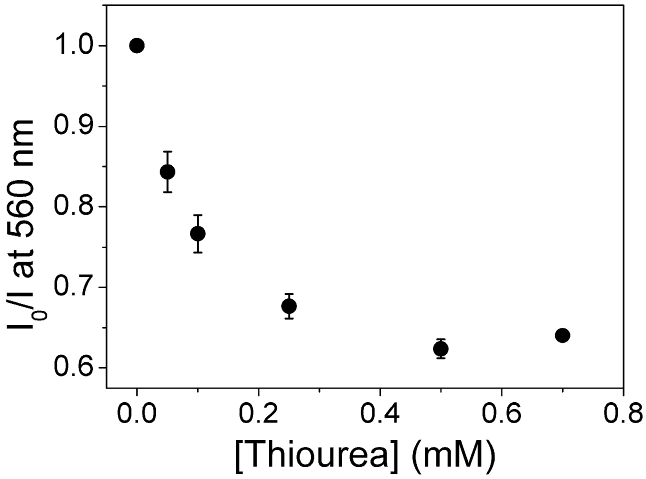

Figure S5. Inhibition efficiency of hydroxyl radical by thiourea in Tris-HCl buffer solution (20 mM, pH 7.4). [PFVCN] = 1.0 × 10^{-6} M, [WS_2] = 1 μg/mL, [Fe^{2+}] = 5 μM, [ssDNA] = 10 nM. The error bars represent standard deviations three parallel measurements. The excitation wavelength is 470 nm.
